# Hyaluronic Acid as a Carrier Supports the Effects of Glucocorticoids and Diminishes the Cytotoxic Effects of Local Anesthetics in Human Articular Chondrocytes In Vitro

**DOI:** 10.3390/ijms222111503

**Published:** 2021-10-25

**Authors:** Lukas B. Moser, Christoph Bauer, Vivek Jeyakumar, Eugenia-Paulina Niculescu-Morzsa, Stefan Nehrer

**Affiliations:** 1 Center for Regenerative Medicine, Department for Health Sciences, Medicine and Research, Danube University Krems, Dr.-Karl-Dorrek-Strasse 30, 3500 Krems, Austria; christoph.bauer@donau-uni.ac.at (C.B.); vivek.jeyakumar@donau-uni.ac.at (V.J.); eugenia.niculescu-morzsa@donau-uni.ac.at (E.-P.N.-M.); stefan.nehrer@donau-uni.ac.at (S.N.); 2 Department of Orthopedics, University Hospital Krems, Mitterweg 10, 3500 Krems, Austria

**Keywords:** hyaluronic acid, glucocorticoids, osteoarthritis, viscosupplementation, intra-articular, injections, LA, chondrotoxicity, biological products, tribosupplementation

## Abstract

The current study aimed to investigate the cytotoxicity of co-administrating local anesthetics (LA) with glucocorticoids (GC) and hyaluronic acid (HA) in vitro. Human articular cartilage was obtained from five patients undergoing total knee arthroplasty. Chondrocytes were isolated, expanded, and seeded in 24-well plates for experimental testing. LA (lidocaine, bupivacaine, ropivacaine) were administered separately and co-administered with the following substances: GC, HA, and GC/HA. Viability was confirmed by microscopic images, flow cytometry, metabolic activity, and live/dead assay. The addition of HA and GC/HA resulted in enhanced attachment and branched appearance of the chondrocytes compared to LA and LA/GC. Metabolic activity was better in all LA co-administered with HA and GC/HA than with GC and only LA. Flow cytometry revealed the lowest cell viability in lidocaine and the highest cell viability in ropivacaine. This finding was also confirmed by live/dead assay. In conclusion, HA supports the effect of GC and reduces chondrotoxic effects of LA in vitro. Thereby, the co-administration of HA to LA and GC offers an alternative less chondrotoxic approach for treating patients with symptomatic osteoarthritis of the knee.

## 1. Introduction

Osteoarthritis (OA) of the knee is a degenerative joint disease characterized by a low-gade joint infection leading to altered chondrocyte metabolism [1]. In a healthy microenvironment, chondrocytes regulate the composition of the extracellular matrix (ECM) such as collagen II and noncollagen proteins (e.g., aggrecan) [2] via a complex signaling network of catabolic and anabolic factors. In OA a metabolic shift of the chondrocytes causes a homeostatic imbalance with increased catabolism and apoptosis and reduced anabolism and autophagy leading to disease progression [3].

Intra-articular injections of local anesthetics (LA) are widely used for treating patients suffering from painful OA of the knee [4]. LA are prevalently administered to OA patients for instantaneous pain relief. The problem is that this effect only lasts a couple of days, depending on pharmacokinetics of the respective substance, and only recurrent injections of LA subside persistent pain relief. However, the cytotoxic effects of LA on chondrocytes are well known [5,6,7]. Intra-articular injection of LA is contra-productive and intensifies the progression of osteoarthritis. The detrimental impact varies depending on the substance being injected [5]. Some studies have shown that ropivacaine induces less apoptosis of chondrocytes in vitro than other anesthetics like lidocaine or bupivacaine [5,8,9]. LA only treat the symptom, namely pain, and accelerates the degenerative process.

In contrast, intra-articular administered glucocorticoids (GC) or hyaluronic acid (HA) target to offset the progression of OA. GC have a potent anti-inflammatory effect which plays a significant role in constraining the progression of OA. However, pro-apoptotic effects of GC on chondrocytes have been reported. This raises the question of whether GC should be administered intra-articular together with local anesthetics because adverse effects on chondrocytes are known for both substances. Interestingly, despite that, their co-administration is frequently performed in clinical practice. HA also has anti-inflammatory effects, albeit to a lesser extent than GC. Advantages of HA include increased cell viability and its role as a viscosupplement for increased lubrication [10]. Furthermore, administered HA induces endogenous HA production [11]. Recently, the co-administration of HA with GC was introduced to combine the beneficial effects of both substances. Primary clinical studies show promising results with decreased pain levels in the co-administration compared to a single injection [12]. Administering different substances offers the potential of achieving synergistic effects and might mitigate detrimental effects of one substance (e.g., cytotoxicity of LA, the apoptotic potential on chondrocytes of GC) by another one (e.g., HA). Onur et al. has shown that co-administration of HA with bupivacaine reduces the cytotoxicity of LA in bovine articular chondrocytes [13], but a similar effect was not observed with lidocaine. The authors have not observed the effects of a co-administrating GC/HA together with LA. Comparatively, studies investigating the effect of this co-administration have not been performed in human articular chondrocytes thus far.

The present study aimed to investigate the cytotoxicity of three different LA (lidocaine, bupivacaine, and ropivacaine) co-administered with either GC, HA, or GC plus HA on human articular chondrocytes in vitro.

## 2. Results

### 2.1. Morphological Examination

**Figure 1:**
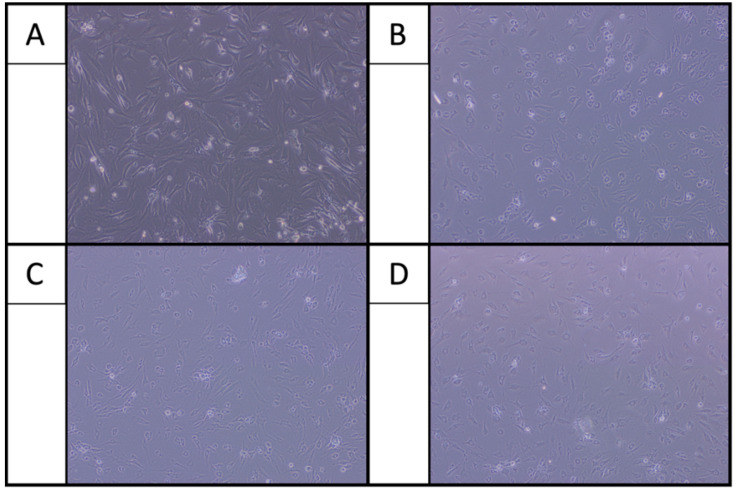
Reduced cell adherence after treatment with LA (**B**) bupivacaine, (**C**) lidocaine and (**D**) ropivacaine compared to the control group in the culture medium (**A**).

**Figure 2:**
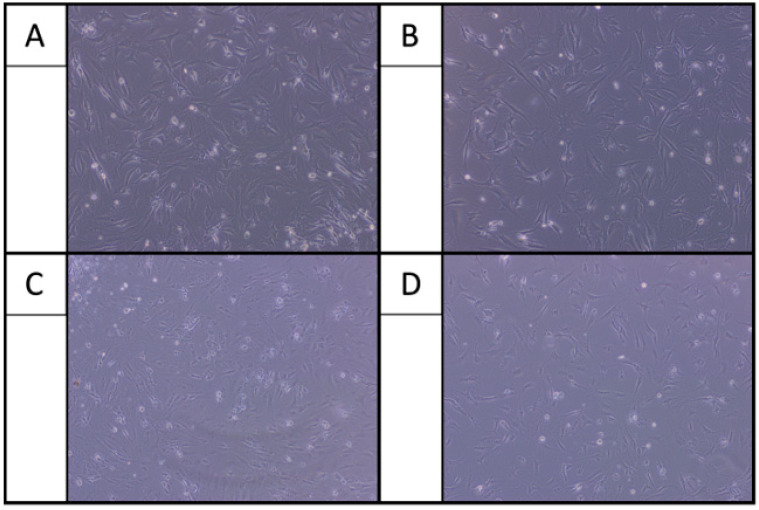
Adding of HA to all LA (**B**) bupivacaine, (**C**) lidocaine and (**D**) ropivacaine resulted in an elongated attachment and branched appearance of the chondrocytes comparable to the control group (**A**).

**Figure 3:**
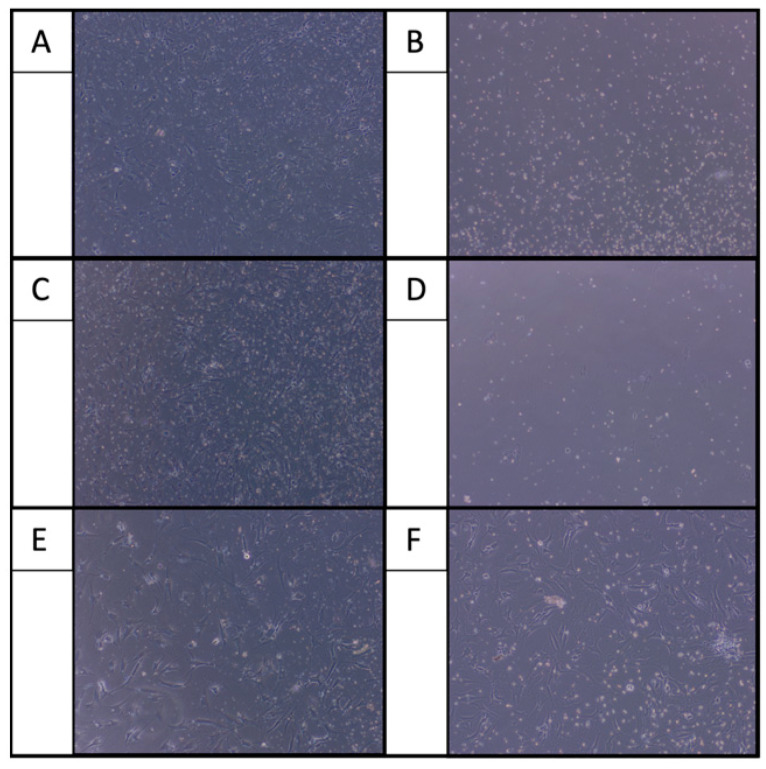
Adding GC to bupivacaine (**B**) and lidocaine (**D**) showed reduced cell adherence. Adding HA to GC (GC/HA) showed better cell adherence for bupivacaine (**A**) and lidocaine (**C**). Ropivacaine with GC (**F**) and GC/HA (**E**) showed best cell adherence.

After 24 h of incubation, osteoarthritic chondrocytes showed elongated fibrochondrocytic appearance in the control group, a characteristic morphology observed on 2D culture substrates. When cells were exposed only to LA, chondrocytes lost this typical morphology and exhibited reduced cell adherence. This finding was independent of the type of LA (Figure 1). Co-administering LA with only HA resulted again in an elongated attachment and branched appearance comparable to the control group. This finding was independent of the type of LA (Figure 2). In contrast, co-administering lidocaine and bupivacaine with only GC showed strongly reduced cell adherence. Adding HA to GC (GC/HA) improved the cell adherence again. Co-administrating ropivacaine with GC/HA showed better cell adherence, comparable to the control group (Figure 3).

### 2.2. XTT Assay

**Figure 4:**
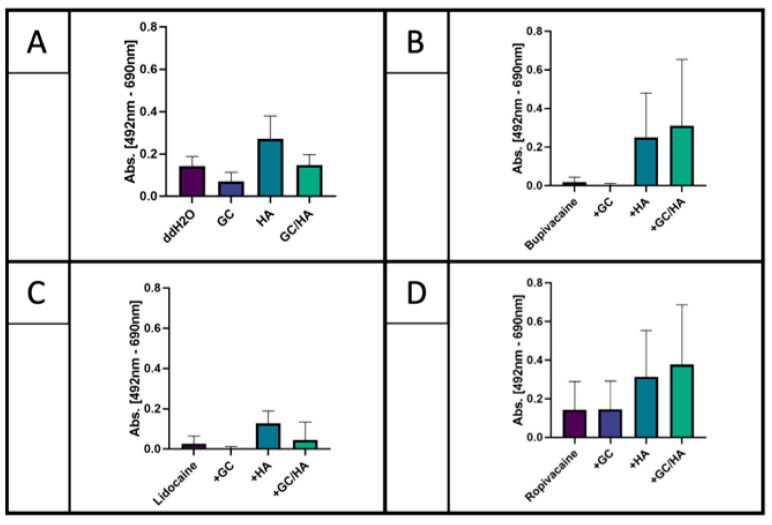
Metabolic activity of test substances in a single dose (**A**) as well as treating cells with LA alone and combined with test substances and LA (**B**) bupivacaine, (**C**) lidocaine, (**D**) ropivacaine. All the results showed no significance between respective groups.

Metabolic activity was inclining in all LA co-administered with HA and GC/HA compared with LA administered alone or combined with GC (Figure 4). Among all LA without any co-administration, ropivacaine exhibited higher metabolic activity. This was on a level comparable to the control or the GC/HA group. Treatment with HA alone elevated metabolic activity, while treatment with GC alone led to a decrease.

### 2.3. Flow Cytometry

**Figure 5:**
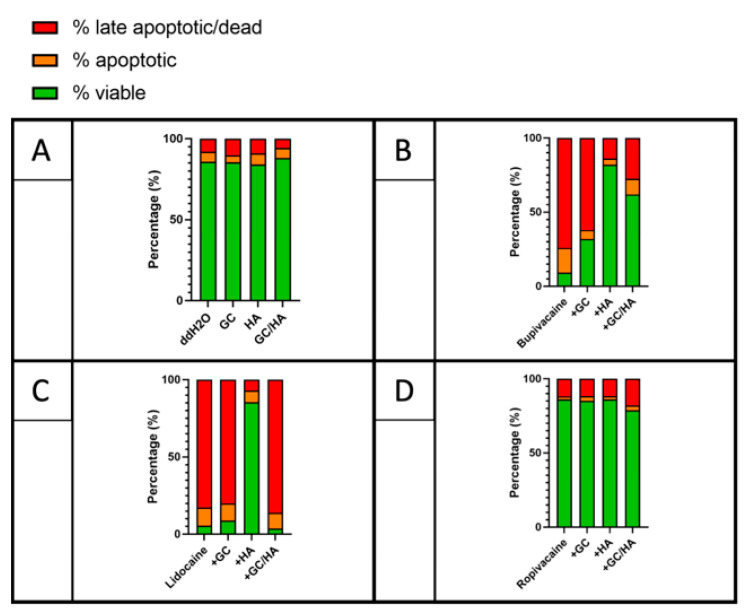
Distribution of viable, early, and late apoptotic chondrocytes in a single dose of the test substances (**A**) and test substances combined with LA (**B**) bupivacaine, (**C**) lidocaine, and (**D**) ropivacaine after 24 h using flow cytometry.

Cell viability was higher in all LA co-administered with HA (lidocaine + HA: 85.66%, bupivacaine + HA: 82.11%, ropivacaine + HA: 86.31%) compared to the other test conditions (only LA, GC, GC/HA) (Figure 5). Significant differences were observed only for lidocaine and bupivacaine. Ropivacaine showed higher cell viability under all test conditions (ropivacaine: 86.17%, ropivacaine + GC: 85.26%, ropivacaine + GC/HA: 78.96%, ropivacaine + HA: 86.31%) without any significant differences. Lidocaine exhibited high early apoptotic and late apoptotic rates in only lidocaine (early apoptotic: 12.00, late apoptotic: 82.50%) and lidocaine + GC (early apoptotic: 11.28%, late apoptotic: 79.86%).

### 2.4. Live/Dead Assay

**Figure 6:**
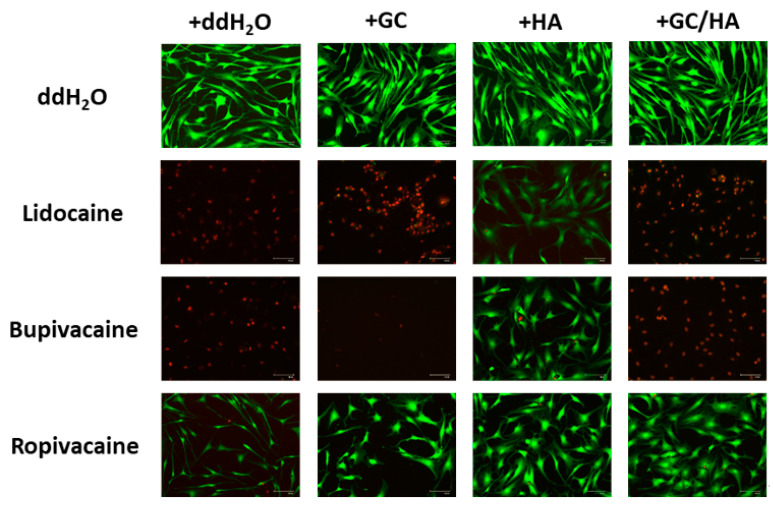
Live/Dead assay shows viable cells in test conditions for local anesthetics plus HA. In other test conditions, lidocaine and bupivacaine led to cell death, while ropivacaine showed viable cells in all test conditions.

Representative images from the live/dead assay showed similar results to flow cytometry (Figure 6). All conditions using test substances (GC, HA, or GC/HA) and ddH_2_O (control group) mainly showed living cells. This was also the case in combination with the local anesthetic ropivacaine. The use of lidocaine and bupivacaine increased cell death under all conditions except with the addition of HA.

## 3. Discussion

The present study aimed to investigate the cytotoxic effect of LA in GC, HA, and a GC/HA mixture on human osteoarthritic chondrocytes in vitro. Subsequently, cells were analyzed for cell viability, cell morphology, and metabolic activity. The most important findings of the present study were the following.

First, the present study confirms the chondrotoxicity of LA, which has been previously reported [14,15]. Morphological analysis using microscopic images showed reduced cell adherence after a one-hour treatment with LA followed by 24 h of incubation in culture medium. Typically, chondrocytes in 2D culture stay attached to the surface with an elongated and branched appearance creating cell–cell interactions. The present study has not shown any difference regarding cell adherence among the different LA. The literature partially supports this finding. Jacob et al. investigated the effect of lidocaine (1% and 2%), bupivacaine (0.5%), and ropivacaine (0.2%) on human chondrocytes cultured in vitro after an incubation period of 30 min under light microscopy [16]. They found an increase in the number of cells with irregular morphology for lidocaine and bupivacaine treatment. Chondrocytes appeared globular and contracted, leading to a disruption in cell–cell interactions. Interestingly, the authors found no irregular morphology in cells treated with ropivacaine. This finding is not in line with the current study’s findings, where reduced cell adherence was also found for ropivacaine. Similarly, Breu et al. assessed the chondrotoxic effects of mepivacaine (2%), ropivacaine (0.75%), and bupivacaine (0.5%) on human articular chondrocytes after a one-hour exposure [5]. Control chondrocytes remained attached with a distinctive morphology, while cells treated with bupivacaine were non-adherent and exhibited aspherical cell morphology without branched appearance. The fraction of non-adherent chondrocytes was distinctly lower after treatment with ropivacaine and mepivacaine. In the present study, metabolic activity decreased in chondrocytes treated with LA and ropivacaine exhibited higher metabolic activity than lidocaine and bupivacaine. The reduced chondrotoxic effects of ropivacaine compared to other LA has already been noted in a review by Piper et al. in 2011 [14]. This finding is also supported by the measurements of flow cytometry in the present study. Most of the cells treated with lidocaine and bupivacaine presented apoptotic (>90%) compared to 86% being viable treated with ropivacaine. Similarly, the live/dead assay has shown mostly living cells treated with ropivacaine and mostly dead cells treated with lidocaine and bupivacaine. This finding is in line with Breu et al., who investigated the chondrotoxic effect of different concentrations of bupivacaine, ropivacaine, and mepivacaine on human cartilage by flow cytometry [5]. In the study of Breu et al., all LA were chondrotoxic, and no significant difference could be observed between them. Although no significant differences were observed among the different LA, the authors concluded that the chondrotoxicity increases from ropivacaine to mepivacaine to bupivacaine. In the present study, lidocaine showed the highest decrease of cell viability which has also been reported by Grishko et al., who investigated the effect of one-hour treatment of LA in different concentrations on human chondrocytes [5]. Here, concentrations of LA like we used in our study (1% lidocaine and 0.5% bupivacaine) led to a detectable and significant decrease in cell viability due to delayed mitochondrial dysfunction and apoptosis. Consequently, they concluded that LA causes chondrotoxicity by mitochondrial DNA damage, leading to chondrocyte apoptosis or necrosis. A review by Kreuz et al. found out that this cytotoxicity depends on the LA used and increases by time and by the applied dose of the LA. Furthermore, they could show that even a single-dose inter-articular injection of LA impedes chondrocyte metabolism and leads to cell death. The authors concluded that LA should only be performed with low concentrations and only for selected patients [4].

Second, GC administered together with LA show a similar chontrotoxic effect compared with only LA. This is in line with the current literature. Brown et al. and Farkas et al. have even shown even increased cytotoxicity when combining GC with lidocaine and bupivacaine on human chondrocytes [17,18]. Farkas et al. also confirmed this effect with a combination of GC and ropivacaine [18]. In the present study, chondrocytes co-treated with GC and lidocaine or bupivacaine were non-adherent, even less adherent when only treated with LA. The underlying mechanism of the reduced proliferation of cultured chondrocyte could be due to the reduced local production of mediators such as IGF-1 that are important for chondrocyte proliferation [19], as it is known that IGF-1 balances the matrix degradation with proteoglycan synthesis to maintain cartilage homeostasis [20]. The catabolic results of GC are shown by the decreased metabolic activity of the chondrocytes in the present study. Moreover, measurements of flow cytometry and live/dead assay did not show any benefit in co-administering GC together with LA.

Third, HA alone and in the GC/HA mixture has beneficial effects on chondrocytes when co-administered with LA. In the present study, improved cell adherence was observed after co-administration of HA with all LA. It is already known that HA enhances the ability of substrate adhesion of chondrocytes, and the proliferative activity of chondrocytes in cell culture has been described as well [21]. The underlying mechanism has been described by Ishida et al., who found that the surface marker CD44 on chondrocytes plays not only a key role in anchoring the extracellular matrix components through binding HA. More importantly, the CD44-HA pathway is also involved in chondrocyte proliferation and matrix production via inducing various stimulatory signals [22]. Taking a closer look at chondrocytes co-administered with GC/HA revealed that the addition of HA improves the cell morphology for bupivacaine and lidocaine. Metabolic activity of chondrocytes elevated when LA were co-administered with HA or GC/HA compared to GC alone. While it has long been believed that the clinical benefits of intra-articular administered HA injections are a result of improved joint lubrication, many studies have also shown physiochemical and pharmacological responses of HA [23,24,25]. Exogenous HA is also incorporated into articular cartilage, having a stimulatory effect on chondrocyte metabolism [11]. This stimulatory effect of HA mitigates the detrimental effects of LA and GC on chondrocyte metabolism in the present study. Live/Dead assay confirms those findings by mostly viable cells when co-administrated with LA and HA. Less dead cells were found when co-administrated with LA plus GC/HA compared to only LA and only GC. Onur et al. investigated the effect of co-administrating HA with LA on bovine chondrocytes [13]. Reduced viability was observed for all conditions compared to media alone (all *p* < 0.003). Chondrocytes treated with bupivacaine + HA showed a high cell viability compared to bupivacaine alone. This positive effect of HA was not observed for lidocaine which is not in line with the present study. The authors concluded that co-administering HA with bupivacaine might protect articular chondrocytes from local anesthetic-associated death. However, the effect of HA on GC and LA was not investigated by Onur et al. or any other author to the best of our knowledge. The present study showed that HA in the GC/HA formulation alleviates the adverse effects of bupivacaine and GC regarding cell viability. This was not the case for lidocaine (low viability) and ropivacaine (high viability in all conditions).

The possible synergistic effect of the investigated substances is of particular importance in treating patients with symptomatic knee OA. LA and GC are still widely used in clinical practice, although the detrimental chondro-destructive effects have been previously recognized and are confirmed by the present study [4]. Despite studies showing the benefits of intra-articular treatment with GC and HA, major international societies disagree with their recommendations considerably [26,27,28,29,30]. In 2013 the American Academy of Orthopedic Surgeons published an inconclusive recommendation for the use of GC in treating patients with knee OA due to the lack of evidence and the unclear balance between benefits and potential risks [27]. Concerns include the increased risk of postoperative infection [31] and cartilage loss due to the catabolic effects of GC on cartilage [29]. In contrast, other major international societies contradict and recommend using GC, which underlines the controversy discussion [28,30,32]. Clinical recommendations for the use of HA or GC/HA for knee OA are less favorable for HA and even more controversy than for glucocorticoids [27,28,30,32]. Thus, there are currently no specific recommendations for the use of HA. While clinical recommendations are mainly based on systematic reviews [33,34] or meta-analysis [35] of clinical studies, and in best case placebo-controlled trials, little is still known about the effects of co-administration of LA plus GC/HA on a cellular level. This is where the present study fills the gap. Recently the use of autologous blood products such as platelet-rich plasma (PRP) has become very popular in the orthopedic field [36]. Platelets comprise anabolic growth factors and anti-inflammatory cytokines that positively effects the osteoarthritic state by inducing cellular proliferation, migration, differentiation and synthesis of the ECM [37]. However, the effect of PRP on chondrogenesis is still controversial and a lack of standardization of PRP preparations makes it difficult to determine the efficacy of PRP [26]. The effects of PRP and HA in combination with LA on human osteoarthritic articular chondrocytes would have been interesting but were not investigated in the present study.

The present study has further limitations. Firstly, the present study was an in vitro study, making it challenging to transfer the findings to physiological conditions. Mimicking a physiological environment in 3D cultures with mechanical stimulation would have been an option to adapt to the in vitro setting. Nevertheless, the present study used human articular chondrocytes from patients undergoing total knee arthroplasty due to knee osteoarthritis. The aim was to mimic the clinical setup for patients undergoing intra-articular infiltration as accurately as possible. Secondly, the present model only investigates short-term effects on chondrocytes. Thirdly, the present study does not consider other components, such as synovial fluid, meniscus, subchondral bone, which is a common limitation in 2D-cell culture. It is well known that chondrocytes lose their chondrocyte phenotype and change to fibroblast-like cell in 2D culture. Human articular chondrocytes require expansion and cell growth for optimal testing conditions due to low cell number. However, cell passaging was only performed once to maintain the chondrocyte phenotype as good as possible. Nevertheless, maintenance of chondrocyte phenotype was not checked in the present study, which is clearly a limitation. Finally, gene expression of cartilage-specific genes was not analyzed to investigate molecular biological effects on the chondrocytes.

## 4. Materials and Methods

### 4.1. Isolation and Cultivation of Human Chondrocytes

Human articular cartilage was obtained from patients undergoing total knee arthroplasty due to degenerative osteoarthritis. The selection process was performed on the following inclusion criteria: (a) Patients suffering from end-stage OA of the knee for one to six years; (b) End-stage OA was radiologically confirmed; (c) The patient obtained informed consent. Exclusion criteria were as follows: (a) Patients receiving anti-inflammatory drugs less than six months before surgery; (b) Patients with operative cartilage treatment of the respective knee; (c) Samples with low chondrocyte cell numbers were excluded. In total, five patients met the inclusion criteria. All patients gave their consent for participating in this study and the regional ethical committee approved the study (GS1-EK-4/665-2020).

Cartilage was stored after surgery in a container with PBS. Transport to the research facility was executed within 24 h. For chondrocyte isolation, articular cartilage was minced into 2 mm^3^ small pieces followed by enzymatic digestion with Liberase TM (0.2 WU/mL, Roche Diagnostics GmbH, Mannheim, Germany) in medium (GIBCO^®^ DMEM/F12 GlutaMAX ™-I, Invitrogen, LifeTech Austria, Vienna, Austria) supplemented with antibiotics (penicillin 200 U/mL; streptomycin 0.2 mg/mL and Amphotericin B 2.5 μg/mL (Sigma-Aldrich Chemie GmbH, Steinheim, Germany) at 37° for 18 to 22 h. Undigested debris was removed under permanent agitation, passing the chondrocyte suspension through a Cell Strainer with 40 μm pores (BD, Franklin Lakes, NJ, USA). Resulting cell pellet after centrifugation (10 min, 500× *g*, room temperature [RT]) was washed with PBS, centrifuged again (10 min, 500× *g*, room temperature [RT]) and resuspended in growth medium with antibiotics, 10% FCS (PAA Laboratories GmbH, Linz, Austria) and 0.05 mg/mL ascorbic acid (Sigma-Aldrich Chemie GmbH, Steinheim, Germany). The viability of the chondrocytes was determined with trypan blue staining (Sigma-Aldrich Chemie GmbH, Steinheim, Germany). Cells were counted using a hemocytometer. For cell expansion, the isolated cells were seeded at a density of 1 × 10^4^ cells/cm^2^ in growth medium in 75 cm^2^ culture flasks (Nunc, Rochester, NY, USA). Cultivation was performed at 37 °C in an incubator with 5% CO_2_. Growth medium was changed every two to three days aiming confluency of 80%. Cells were then harvested using accutase (1.5 mL/flask; PAA Laboratories GmbH, Linz, Austria). Cell count was performed as described above, and the cells were seeded in 24-well plates at a density of 1 × 10^4^ cells/cm^2^ and incubated in 500 µL culture medium at 37° and 5% CO_2_ for three days to ensure adherence of cells to the surface.

All products are commercially available and widely used in clinical practice. In total, 16 conditions of various combinations were investigated concerning their cytotoxicity. The LA (Lidocaine 1%, Bupivacaine 0.5%, Ropivacaine 2%) and the test substances (Glucocorticoid triamcinolone hexacetonide [4.5 mg/mL], hyaluronic acid [22 mg/mL] and the combination of both) were 1:1 diluted with ddH_2_O to test their single effect on the chondrocytes. Moreover, all three LA were additionally diluted 1:1 with each of the test substances to perform cytotoxicity tests. Finally, adding ddH_2_O to the cells was used for control purposes.

### 4.2. Treatment of the Cells

After three days of incubation, the culture medium was discarded, while chondrocytes were treated with 500 µL of the various conditions for 1 h in the incubator (37 °C, 5% CO_2_). After 1 h, the substances were removed, and cells were rinsed twice with culture medium. Finally, 500 µL of culture medium was added to each well and further incubated for 24 h. After 24 h, the morphology of the chondrocytes was imaged. Similarly, metabolic activity and flow cytometry were performed to analyze cell viability and apoptosis.

### 4.3. Morphological Examination

Cell morphology of each test condition was examined after the 24-h incubation cycle by taking microscopic images of two different regions of the 24-well using a phase-contrast microscope.

### 4.4. Metabolic Activity

The metabolic activity of chondrocytes was measured using an XTT-based ex vivo toxicology assay kit according to the manufacturer’s instructions (Cell Proliferation Kit II, Roche Diagnostics, Basel, Switzerland). XTT labeling mixture was prepared using 245 µL electron-coupling reagent and 5 µL XTT labeling reagent for a single reaction. XTT labelling mixture (250 µL) was added to the culture medium of each well and incubated for 4 h at 37 °C and 5% CO_2_. After incubation, the absorbance was measured at 492 nm and 690 nm (background wavelength) using a multi-mode microplate reader (Synergy 2, Winooski, VT, USA) with Gen 5 software.

### 4.5. Flow Cytometry

Flow cytometry was performed to analyze the distribution of live and apoptotic chondrocytes following the exposure of the test substances. Chondrocytes were detached by the use of accutase after 24 h and counted. Cell suspension from each condition was centrifuged (439× *g* for 10 min), the cell pellet was washed once with PBS and centrifuged again. The supernatant was discarded, and the cell pellet was resuspended in 200 µL of 1X annexin V binding buffer. Staining with 7-AAD (late apoptosis, dead cells) and annexin V (early apoptosis) phycoerythrin (PE) was performed using a PE Annexin V Apoptosis Detection Kit I according to the manufacturer’s instructions (BD Biosciences, Franklin Lakes, NJ, USA). Flow cytometry analysis was executed using a Gallios flow cytometer (Beckman Coulter, Brea, CA, USA) equipped with 405 nm, 488 nm, and 638 nm lasers and Kaluza Analysis Software (Beckman Coulter, Brea, CA, USA), which was used for measurements and data analysis.

### 4.6. Live/Dead Assay

The supernatant was aspirated, and cells were washed once with DPBS. Next, DPBS was mixed with a 2 mM Ethidium homodimer-1 (EthD-1) stock solution to reach a 4 µM EthD-1 solution. Then calcein AM stock solution (4 mM) was added for a 2 µM calcein AM working solution. The combined reagents were mixed and pipetted into the well-plate. Cells were incubated for 20 min at 37 °C in the incubator before the live/dead solution was discarded. Cells were washed twice with DPBS and imaged using an EVOS FLoid Cell Imaging Station (Life Technologies, Carlsbad, CA, USA).

### 4.7. Statistical Analysis

Data values are reported as the mean ± standard deviation. Significant level was set at *p* < 0.05. Multiple comparisons were performed using nonparametric Kruskal–Wallis test followed by Dunn’s multiple comparisons test. Statistical analysis was performed using the GraphPad Prism software (Graphpad Prism Software Inc., San Diego, CA, USA).

## 5. Conclusions

The study showed that co-administering LA with HA and HA/GC reduces its chondrotoxicity. Administering GC is desirable due to its potent anti-inflammatory effect, albeit its apoptotic potential is problematic. In the present study, HA supports the effects of GC and reduces the cytotoxic effects of LA. Differences between LA were observed; (i) lidocaine showed high apoptotic rates; (ii) ropivacaine showed the best cell viability. The findings of the present study are of high clinical relevance. Although the chondrotoxic effect of LA and GC is well known, the substances are widely used in a clinical setting. Our data suggest that co-administrating HA with LA and GC might mitigate their detrimental effects on chondrocytes while still maintaining the beneficial effects of anti-inflammation. Clinical studies will be necessary to find out whether the beneficial painkilling properties are impaired by this co-administration.

## Data Availability

Not applicable.
